# Correlates of the Women’s Development Army strategy implementation strength with household reproductive, maternal, newborn and child healthcare practices: a cross-sectional study in four regions of Ethiopia

**DOI:** 10.1186/s12884-018-1975-y

**Published:** 2018-09-24

**Authors:** Zufan Abera Damtew, Ali Mehryar Karim, Chala Tesfaye Chekagn, Nebreed Fesseha Zemichael, Bantalem Yihun, Barbara A. Willey, Wuleta Betemariam

**Affiliations:** 1 Federal Ministry of Health of Ethiopia, Health Extension and Primary Health Service Directorate, Sudan Street, Addis Ababa, Ethiopia; 2 Last 10 Kilometers Project (L10K) 2020, JSI Research & Training Institute, Inc, Bole Sub-City, Kebele 03/05, Hs #, 2111 Addis Ababa, Ethiopia; 3 Department of Infectious Disease Epidemiology, Faculty of Epidemiology and Population Health, London School of Hygiene and Tropical Medicine, Keppel Street, London, WC1E 7HT UK

**Keywords:** Ethiopia, Community health workers, Health extension workers, Women’s development Army, RMNCH

## Abstract

**Background:**

To address the shortfall in human resources for health, Ethiopia launched the Health Extension Program (HEP) in 2004, establishing a health post with two female health extension workers (HEWs) in every *kebele* (community). In 2011, the Women’s Development Army (WDA) strategy was added, using networks of neighboring women to increase the efficiency of HEWs in reaching every household, with one WDA team leader for every 30 households. Through the strategy, women in the community, in partnership with HEWs, share and learn about health practices and empower one another. This study assessed the association between the WDA strategy implementation strength and household reproductive, maternal, newborn and child health care behaviors and practices.

**Methods:**

Using cross-sectional household surveys and community-level contextual data from 423 kebeles representing 145 rural districts, an internal comparison group design was applied to assess whether HEP outreach activity and household-level care practices were better in kebeles with a higher WDA density. The density of active WDA leaders was considered as WDA strategy implementation strength; higher WDA density in a kebele indicating relatively high implementation strength. Based on this, kebeles were classified as higher, moderate, or lower. Multilevel logit models, adjusted for respondents’ individual, household and contextual characteristics, were used to assess the associations of WDA strategy implementation strength with outcome indicators of interest.

**Results:**

Average numbers of households per active WDA team leader in the 25th, 50th and 75th percentiles of the kebeles studied were respectively 41, 50 and 73. WDA density was associated with better service for six of 13 indicators considered (*p* < 0.05). For example, kebeles with one active WDA team leader for up to 40 households (higher category) had respectively 7 (95% CI, 2, 13), 11 (5, 17) and 9 (1, 17) percentage-points higher contraceptive prevalence rate, coverage of four or more antenatal care visits, and coverage of institutional deliveries respectively, compared with kebeles with one active WDA team leader for 60 or more households (lower category).

**Conclusion:**

Higher WDA strategy implementation strength was associated with better health care behaviors and practices, suggesting that the WDA strategy supported HEWs in improving health care services delivery.

**Electronic supplementary material:**

The online version of this article (10.1186/s12884-018-1975-y) contains supplementary material, which is available to authorized users.

## Background

Despite progress over the last two decades, improvements in maternal and newborn survival have not been felt equally across the globe, and low and middle-income countries continue to be disproportionately affected, experiencing 99% of maternal deaths [1], with 66% in Sub-Saharan African countries [2]. Furthermore, within countries, mortality is higher among women and newborns living in rural areas, and among those from poorer communities [3]. Ethiopia reported a neonatal mortality rate of 28 per 1000 live births [4] and a maternal mortality ratio of 353 per 100,000 live births in 2015 [1]. Although these represent declines of 38 and 43% respectively from 2003 levels [5], maternal mortality in Ethiopia remains over 20 times higher than in European countries [1]. The majority of maternal and newborn deaths worldwide could be prevented by providing quality antenatal, delivery, postpartum and postnatal care [1]. Estimates for Ethiopia suggest improved uptake of professional care, but access remains well below levels required, with 32% of pregnant women having the recommended four antenatal care (ANC) visits, 28% skilled attendance at delivery and 17% of mothers having a postpartum check within two days of delivery [6, 7]. Furthermore, marked inequalities exist, for example data from the 2016 Ethiopia Demographic and Health Survey (DHS) show that although 80% of women living in urban areas experienced skilled attendance at delivery, only 21% of pregnant women living in rural areas did so [7].

Reasons for low access to quality services are multi-faceted; however, challenges of insufficiently skilled health workers and inequitable distribution of the health workforce play a role [8]. This is especially the case in countries like Ethiopia, where a large population live in rural areas with low health worker per population density. In total, over 80% of the population of Ethiopia live in rural areas [9]. The ratio of available health workers (including: doctors, health officers, nurses and midwives) is 7 per 10,000 population [9], far below the World Health Organization-recommended minimum threshold of 23 per 10,000 population required to provide basic health coverage [10]. Numerous countries have responded to this growing challenge with the creation and deployment of community health workers, an alternative health worker cadre who can deal effectively with common health problems and improve access to services [11, 12].

Community health workers can contribute to maternal health and child survival through basic curative care and preventive services, and act as a conduit to professional care [13, 14]. The incorporation of community health workers has a long history in public health, and national-scale programs exist in a number of low and middle-income countries. For example, in the late 1970s the Ghanaian Ministry of Health introduced substantial numbers of community or village health workers aimed at implementing primary health care strategies [15]. In 1982, Indonesia deployed village health volunteers, selected and paid by local communities. Their activities included family planning, health education, growth monitoring, nutrition support, immunization and treatment, particularly of diarrheal diseases [16]. In 1988, the Female Community Health Volunteer Program was established by the Nepalese Government and covers most of the country, with over 48,000 volunteers [17]. In 1994, Pakistan implemented the Lady Health Worker Program as part of a national strategy to reduce poverty and improve health by bringing health services to the doorsteps of underserved communities [18].

The effectiveness of such community health worker programs for delivering disease preventive and basic curative services has generally been positive, and in some contexts contributed to declines in maternal, infant and child mortality, although the extent and quality of the evidence available for low and middle-income countries is limited [19, 20]. A recent systematic review from Gilmore and McAulifee reported on the effectiveness of community health worker programs with a focus on preventative strategies to improve reproductive, maternal, newborn and child health (RMNCH) in 10 low and middle-income countries [21]. Based on 17 studies, the authors concluded that, although additional evidence for some strategies was needed, common packages included health education, breastfeeding promotion, essential newborn care and psychosocial support, and that strategies promoting mother-performed behaviors (e.g. skin-to-skin contact for thermal care) were especially effective.

In Ethiopia, the Health Extension Program (HEP) is a community-based health service delivery system, launched in 2004 to provide equitable access to promotive, preventive and limited curative health interventions, with emphasis to the needs of the majority, less-privileged, rural population [22]. In each *kebele*, (community) a health post was established, staffed by two salaried female Health Extension Workers (HEWs) who had graduated from high school and undergone one year’s training. The Ethiopian Federal Ministry of Health’s HEP aims to provide primary health services through one HEW for every 2500 people. Since its inception, over 40,000 HEWs have been deployed in rural, pastoral and urban areas, and more than 16,500 health posts have been built across the country to provide and manage primary health care [23]. The HEP provides 16 health service packages categorized in four essential health components: hygiene and environmental health services (e.g. encouraging the construction and use of latrines), family health services (e.g. antenatal care (ANC)), disease prevention and control (e.g. malaria prevention and control), and health education and communication (Additional file 1: Table S1). The design of the HEP packages was based on analysis of the major health problems and disease burdens of the population. Recently, additional services were included, particularly for children and women, such as Integrated Community-Based Case Management, Community-Based Newborn Care, and the provision of long-acting family planning methods.

**Fig. 1:**
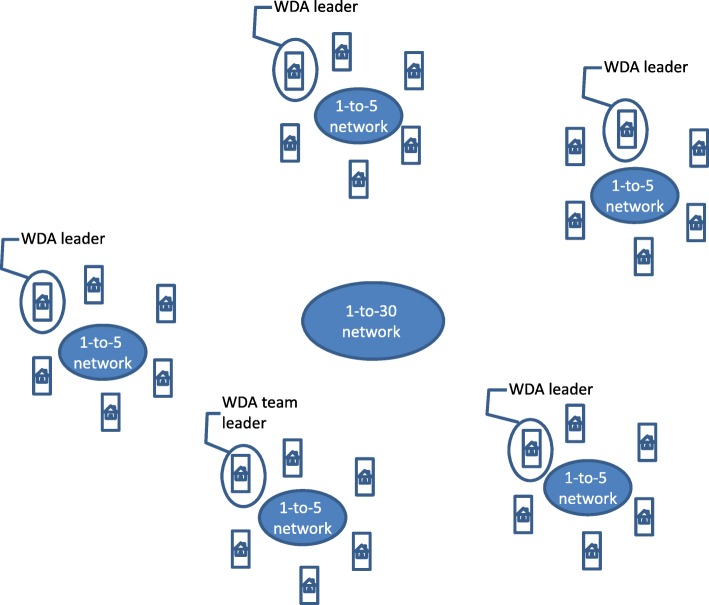
The Women’s Development Army 1–30 network. The Women’s Development Army 1–30 network is organised in the community, with five participating neighboring households creating a women’s development team, and groups of five or six teams brought together as a network, under one leader

The HEP has brought impressive results: large reductions in maternal and child mortality, and morbidity and mortality by major communicable diseases, through providing basic health services and bridging the gap between the community and health facilities [9]. However, to enhance these efforts and improve geographical penetration and equity, in 2011 the Ethiopian Government introduced a complementary initiative, the Women’s Development Army (WDA) strategy, also known as the Health Development Army. WDA members are organized by their neighborhood and are commonly called “one-to-five” networks (denoting one leader and five member households). Based on the neighborhood, five or six “one-to-five” networks are grouped into a women’s development team that comprises 25 to 30 households, called “one-to-30” (denoting one team leader to about 30 member households) (Fig. 1). The WDA is a systematic, organized, inclusive and collaborative movement of neighboring households. The WDA leaders of the 1-to-5 networks receive initial training from a HEW, with technical support from the health center staff, on the 16 key health packages, which form the core of the HEP. Under the guidance of HEWs and community leaders, all community members participate, teach and learn from each other and take practical actions to improve the health of individuals, families and the community. The WDA team leaders support the HEWs by mobilizing the community to take up key health services, disseminating essential health messages to the communities, practicing key health actions and collecting health information on community members eligible for health services. This approach helps to increase community engagement and health seeking behavior.

The activities of community health workers are expected to lead to enhanced demand for care and improved home-based practices. For example, enhanced demand for ANC and facility-based delivery care; improved home-based maternal and newborn care practices, such as thermal care; enhanced frequency and timing of postpartum home visits; and increased coverage of immunization [14].

This paper is the first of a series of four investigating innovations designed to support the HEP and the WDA strategy, to improve maternal and newborn health in Ethiopia. The other three papers consider Community-Based Data for Decision-Making [24], the use of a Participatory Community Quality Improvement strategy [25], and a Family Conversation strategy [26]. The WDA strategy has been reported to enhance the demand and access of the community to the health services [23, 27], but there is a lack of evidence isolating the contribution of the WDA strategy in enhancing maternal and child health care outcomes. This study therefore aimed to examine the relationship between the density of active one-to-30 network leaders and uptake of RMNCH services in 145 rural districts, in four regions of Ethiopia. Although the WDA are taking part in a variety of developmental activities, including the implementation of the HEP more widely, the focus of this study is on the contribution of the WDA and HEWs to improving maternal, neonatal and child health.

## Methods

### Settings

Administratively, Ethiopia is composed of nine regional states: Tigray, Afar, Amhara, Oromia, Somali, Southern Nation Nationalities and Peoples’ (SNNP) Region, Benishangul-Gumuz, Gambella, and Harari; and the city administrative councils of Dire Dawa and Addis Ababa. Projections from the 2007 population and housing census estimate the total population in 2017 to be more than 95 million, with the majority of the population (over 80%) residing in rural areas [28]. The availability of health workers — doctors, health officers, nurses and midwives — to population ratio is 0.7 per 1000 population, far behind the minimum threshold of 2.3 doctors, nurses and midwives to 1000 population ratio required to ensure high coverage with essential health interventions [29]. This study was conducted in four most populous regions of Ethiopia: Oromia, Amhara, Tigray and SNNP. According to the Centeral Statistical Agency population projection, these four regions account 86% of the total population. Including health posts, 18,107 health facilities are providing services in these regions [30].

The WDA strategy was introduced in 2011 with the objective of supporting the HEP to reach households and communities with actionable messages. Since 2008, the Last Ten Kilometers project (L10 K) has also been supporting the HEP. In partnership with 12 local civil society organizations, L10 K has been implementing innovative community-based strategies primarily aimed at improving utilization of high impact maternal, newborn, and child health services through strengthening the HEP and the WDA strategy. Details of the L10 K intervention strategies are provided in Additional files 1, 2 and 3.

### Study design

Since the WDA strategy was implemented across the four most populous regions of Ethiopia; Amhara, Oromia, SNNP and Tigray, using a conventional intervention comparison group design was not possible. Instead, we constructed an internal comparison group by taking advantage of the natural variability in the intensity of WDA strategy implementation across 423 kebeles. Accordingly, we assessed whether HEP outreach activity and household RMNCH care behaviors and practices were better in kebeles with comparatively better implementation of the WDA strategy with active WDA leaders.

### Study participants and data collection

We used data from a cross-sectional household and health post survey, conducted from December 2014 to January 2015 (Additional file 1). The survey was a two-stage cluster survey, stratified by region and program domain, in 145 *woredas* (districts), of which 115 were in the L10 K project intervention (L10 K Platform) area and 30 were not. We used data from three target groups: i) family planning information from women aged 15 to 49 years; ii) maternal and newborn health information from women with children 0 to 11 months of age; and iii) childhood immunization information from women with children 12 to 23 months of age. At the first stage, kebeles were selected as primary sampling units with probability proportional to their estimated population sizes, stratified by region and by whether or not they were in the L10 K Platform area. At the second stage, the 30 by seven cluster survey strategy, commonly used for monitoring the coverage of childhood immunization services, was adapted to obtain information from the three target groups [31]. In brief, the first household was selected from the middle of the kebele and then every fifth household was visited, moving away from the center, and all the women in that household were interviewed if they were within the target population. If a woman aged 15 to 49 had a child between 0 to 11 months of age, she was interviewed for the women of reproductive age questionnaire and for the questionnaire for women with children aged 0 to 11 months. After completion of the quota for women of reproductive age in a kebele, the interviewers focused on completing interviews for the other target groups. The desired quota for each target group was 12 respondents.

The household survey instruments were translated into the three major local languages (Amharic, Oromifa, and Tigrigna). In the SNNP region, which has 11 additional languages, the interviewers translated from Amharic while administering the questionnaires. Verbal consent was sought and documented by the interviewer. If the respondent was under 18 years old, consent was sought from her husband, parents or guardian. As it was expected that most of the respondents could not read or write, written consent was not sought. Ethical clearance was obtained from the Institutional Review Boards of the respective Regional Health Bureaus and of JSI Research & Training Institute, Inc.

Information on the WDA strategy implementation strength and other contextual factors of a kebele was obtained from health post records and HEW interviews. For this purpose, the health post in the selected kebele was visited and a HEW in the selected health post was interviewed. If more than one HEW was present, they were interviewed together, after seeking their written consent. In the few cases where there was more than one health post in a kebele, one was randomly selected for the HEW interview and the household interviews were conducted from within the administrative boundaries of the selected health post.

### Outcomes of interest

Thirteen indicators were considered as the outcomes of interest. These were grouped into two broad categories: (1) HEP outreach activity indicators, which were measured among unique respondents from all three target groups of women; and (2) RMNCH care practice indicators. The latter were sub-categorized into (i) family planning, which was measured among respondents of reproductive age who were in union; (ii) maternal and newborn health, which included care behavior and practices during the most recent pregnancy as reported by women with children in their first year of life; and (iii) childhood immunization, which was measured among women with children age 12 to 23 months. The definitions of the indicators are given in Table 1.

**Table 1:** Definition of Health Extension Program outreach activity and RMNCH care practice indicators

Indicator	Definition
(1) HEP outreach activity	
Households with latrine	The percentage of households with functioning latrine
Households visited by HEW	The percentage of respondents whose households were visited by HEWs to discuss about health related issues within six months prior to the survey
Households with family health card	The percentage of respondents whose households have Family Health Card. Distributed by HEWs to all women of reproductive age in a household, the cards are used as a tool to provide health education, such as promoting maternal, newborn and child health
(2) RMNCH care practices	
(i) Family planning	
Contraceptive prevalence rate	The percentage of married (or in union) women of reproductive age and/or their partners who were using any method of contraception to delay or avoid getting pregnant during the survey
(ii) Maternal and newborn health	
Received four or more ANC visits	The percentage of women who went to a health facility for antenatal care at least four times during last pregnancy
Neonatal tetanus protected childbirth	The percentage of women whose last childbirth was protected against neonatal tetanus
Delivery at health facility	The percentage of women who gave their last childbirth at a health facility
Early PNC	The percentage of women who were visited by HEWs at home for PNC or newborn care within 48 h of last childbirth
Practiced clean cord care for their newborn	The percentage of women without skilled birth attendance, who cut the umbilical cord of their last newborn with a sterile instrument, tied the cut end of the cord with sterile thread, and applied nothing to the cut end of the umbilical cord
Practiced thermal care for their newborn	The percentage of women without skilled birth attendance, who dried and wrapped their last newborn immediately after birth, delayed bathing the newborn by six hours or more, and always maintained skin-to-skin contact with the baby
Immediate breastfeeding	The percentage of women without skilled birth attendance, but who initiated breastfeeding the newborn immediately after birth
(iii) Childhood immunization	
Children received all vaccines	The percentage of children between 12 and 23 months who received all childhood vaccines
Dropout	The percentage of children between 12 and 23 months who dropped out between Penta 1 and Penta 3 vaccines

*ANC* Antenatal care, *HEP* Health Extension Program, *HEW* Health extension worker, *PNC* Postnatal care, *RMNCH* Reproductive, maternal, newborn and child health

### Independent variables

The independent variable of interest was the WDA density, a measure of the WDA strategy implementation strength. For each kebele, it was defined as the ratio of the number of households in the kebele per active WDA team leader. Active WDA team leaders were those who were recorded by the HEW as having met with her and discussed RMNCH issues within the WDA team leaders’ network during the three months preceding the survey. To facilitate the interpretation of the findings, the kebeles were categorized into three groups of WDA strategy implementation strength: higher, moderate and lower. None of the sampled kebeles had one active WDA team leader for every 30 households as expected by the program. Thus, a higher cut-off point of 40 or less households per active WDA team leader was used to define higher WDA density. Kebeles with less than half the expected WDA density (more than 60 households per active WDA team leader) were categorized as having lower WDA density; while those with between 40 and 60 households per active WDA team leader were categorized as having moderate WDA density. Accordingly, 25% (104), 41% (173) and 35% (146) of the kebeles were categorized as having higher, moderate and lower WDA density respectively.

The independent variables considered in the regression analysis were the individual, household and contextual characteristics of the respondents. The individual-level factors were age, education, marital status and parity; the household-level factors were wealth quintile and distance of the respondent’s household from the nearest health facility; and the contextual factors were HEW to population ratio of the kebele, administrative region and presence of the L10 K Platform.

We included HEW to population ratio of the kebele to account for variations in the input of the HEP across kebeles. As indicated earlier, HEWs are mentors of WDA members; as such, we do not assume that effects of the WDA strategy implementation strength on RMNCH care behaviors and practices are independent of HEWs’ efforts.

The wealth index score was constructed for each household with a principal components analysis of the household possessions (electricity, watch, radio, television, mobile phone, telephone, refrigerator, table, chair, bed, electric stove and kerosene lamp), and household characteristics (type of latrine and water source). The households were ranked according to the wealth score and then divided into five quintiles [32].

### Sample size

The household survey obtained data from 14,049 women in 478 kebeles (Additional file 1); however, the analysis was restricted to the data obtained from 12,381 women in 423 kebeles for which measures of both WDA density and HEW to population ratios were available. Of the 12,381 unique women that were included in this study, 26% (3166) were respondents for at least two of the three target groups; 44% (5397) of respondents were women of reproductive age; 41% (5081) of respondents were women with children aged 0 to 11 months; and 41% (5088) of respondents to the questionnaire were women with children aged 12 to 23 months.

### Statistical analysis

Stata 14.2 was used for statistical analysis [33]. First, whether the individual and household level characteristics of the 2668 respondents from the 55 kebeles that were excluded from the analysis were different from those that were included was assessed using Pearson’s chi-squared statistics adjusted for survey design effect. Similar tests were done to assess whether the individual, household and contextual characteristics of the respondents were associated with WDA strategy implementation strength to examine possible program placement bias (such as whether kebeles with higher WDA densities were associated with other kebele-level factors that influenced the outcomes of interest).

Multilevel analysis using kebele-level random effects logistic regression models were used to assess the associations between WDA strategy implementation strength categories and the outcomes of interest. Stata’s ‘*xtlogit*’ command was used for the purpose. The models were adjusted for survey design and individual, household and contextual characteristics of the respondents. To assess the contribution of the respondents’ characteristics to the model, they were grouped into individual, household and contextual factors. First, the model with all the three groups of independent variables was estimated. Then, the statistical significances of each of the three groups were assessed one by one, using likelihood ratio test. If a particular group was not statistically significant at *p* < 0.05, it was dropped. The goodness-of-fit of the models were assessed using the global Wald’s statistics, likelihood ratio test of the kebele-level random effects, and sensitivity of the quadrature approximation that was used to estimate the models. Once the final model was identified for each outcome, Stata’s post-estimation ‘margins’ command was applied to obtain the adjusted estimates of the outcome of interest indicators according to the WDA density categories, holding the kebele-level random effects constant. Then, the percentage-point differences of the adjusted estimates between moderate and lower WDA density and between higher and lower WDA density, with its 95% confidence intervals, were estimated using nonlinear combinations of estimators. Statistical significance of the associations between exposure and the outcomes of interests were concluded if the 95% confidence intervals of the percentage-point differences did not overlap the value zero.

## Results

The community health structure of Ethiopia comprises the HEP, with HEWs supported by the WDA [9, 23, 27]. The sample characteristics of the 2668 respondents from the 55 kebeles that were excluded from the analysis were not statistically significantly different from those that were included (*p* > 0.1; analysis not shown).

The average number of households per active WDA leader in the 25th, 50th and 75th percentiles of the kebeles in the study area were 41, 50 and 73 respectively (analysis not shown). Table 2 describes the sample characteristics of unique respondents and their association with WDA density categories. Nearly half (47%) of the respondents were aged 25 to 34 years; most (60%) could not read; 93% were married; most (59%) had three or more children; more than half (54%) lived within 30 min of a health facility; 84% lived in the L10 K intervention area. About 44% of respondents were from kebeles where fewer than 2500 people received services from one HEW (in line with Ethiopian Federal Ministry of Health guidelines), whereas around 45% were from kebeles where one HEW was serving 2500 to 5000 people, and the remaining 11% were from kebeles where one HEW was serving more than 5000 people. Interestingly, women in the lowest wealth quintile were more likely to be resident in kebeles with higher WDA density (*p* < 0.05). In total, 27% of women in the lowest wealth quintile were resident in a kebele with higher WDA density compared with 14% of women in the highest wealth quintile.

**Table 2:** Percentage distribution of the sample characteristics by WDA density

Characteristics	Category	WDA density			Total	*p-value
		Lower	Moderate	Higher		
Age group	15–19	9	9	9	9	0.574
	20–24	22	23	23	22	
	25–34	46	49	46	47	
	35–49	23	20	22	22	
Education	Cannot read	60	58	62	60	0.336
	Primary	22	22	23	22	
	Higher	18	20	16	18	
Marital status	Other	6	7	7	7	0.319
	Married/in union	94	93	93	93	
Number of children	0	4	5	4	5	0.151
	1	21	20	24	22	
	2	15	15	15	15	
	3	13	14	15	14	
	4+	46	45	42	45	
Wealth quintile	Lowest	18	18	27	20	0.008
	Second	19	19	24	20	
	Middle	20	21	18	20	
	Fourth	22	20	19	20	
	Highest	22	22	14	20	
Distance to any health facility	< 30 min	52	55	55	54	0.493
	30 to 59 min	33	28	31	30	
	1+ hours	16	17	14	16	
Religion	Orthodox	50	55	75	58	0.005
	Protestant	26	17	12	19	
	Muslim	24	27	13	22	
	Other	1	2	0	1	
Region	Tigray	12	17	25	18	< 0.001
	Amhara	24	26	46	30	
	Oromia	26	25	21	25	
	SNNP	38	31	9	28	
L10 K area	No	17	12	22	16	0.115
	Yes	83	88	78	84	
HEW density (population per HEW in *kebele*)	2499	39	44	49	44	0.690
	2500 to 3499	30	29	26	29	
	3500 to 4999	15	17	17	16	
	5000+	16	10	8	12	
Women of reproductive sample	No	56	55	57	56	0.305
	Yes	44	45	43	44	
Women with children 0 to 11 months sample	No	59	60	58	59	0.205
	Yes	41	40	42	41	
Women with children 12 to 23 months sample	No	59	60	59	59	0.076
	Yes	41	40	42	41	
Number of *kebele*		147	173	103	423	
Number of respondents		4249	5135	2997	12,381	

*HEW* Health extension worker, *SNNP* Southern Nations, Nationalities and Peoples, *WDA* Women’s Development Army

*p-values are for the chi-square statistics testing the null hypothesis that the sample characteristics are similar between the different levels of WDA density categories

Among the sample characteristics, wealth quintile, religion and administrative regions were statistically significantly associated with WDA density (*p* < 0.05), indicating the possibility of program placement bias (Table 2). The majority (51%) of the respondents from kebeles with higher WDA density were from the lowest two wealth quintiles; indicating that WDA strategy implementation strength was pro poor. The proportion of respondents from Amhara region was comparatively high in kebeles with higher WDA density (46%), compared to from moderate (26%) or lower (24%) density kebeles. A similar trend was observed in respondents from Tigray region; while the opposite was observed in respondents from Oromia and SNNP regions. The proportion of respondents who were Orthodox Christians was higher in higher WDA density kebeles (75%) than those from moderate (55%) or lower WDA density (50%) kebeles. The association between religion and WDA density was not surprising since almost all the respondents from Tigray (97%) and Amhara (99%) regions were Orthodox Christians (analysis not shown).

Multilevel logit models were estimated to assess the associations between the WDA density categories and the outcomes of interest. The coefficients of the multilevel models and their goodness-of-fit statistics (global Wald’s statistics) are provided in an additional table (Additional file 4). All three groups of respondents’ characteristics were statistically significant (*p* < 0.05) in all the final models. The kebele-level random-effects was statistically significant (*p* < 0.001) for all the models estimated; thus indicating the appropriateness of the multilevel analysis.

Adjusted estimates of the outcomes of interest indicators according to the WDA density category were estimated from the multi-level models and presented in Table 3. The percentage-point differences of the adjusted estimates between moderate and lower WDA density and between higher and lower WDA density categories, with 95% confidence intervals, are also presented. The analyses indicate that all three HEP outreach activities’ indicators were higher in kebeles where the WDA density was in the higher category (Table 3). Households with a latrine were respectively 12 (95% Confidence Interval: 7, 16) and 14 (95% CI: 9, 18) percentage-points higher in moderate and higher WDA density areas compared with lower WDA density areas. Similarly, households visited by HEWs were 7 (95% CI: 3, 11) and 9 (95% CI: 4, 14) percentage-points higher in moderate and higher WDA density areas compared with lower WDA density areas. While households with Family Health Cards were 11 (95% CI: 6, 16) percentage-points higher in higher WDA density areas compared with lower WDA density areas.

**Table 3:** Adjusted estimates of the outcome indicators by WDA density

HEP outreach activities and RMNCH indicators	Total (%)	WDA density			%-points difference (95% CI)	
		Lower (%)	Moderate (%)	Higher (%)		
					Moderate and Lower	Higher and Lower
HEP outreach activity						
Households with latrine	78	70	81	83	12 (7, 16)	14 (9, 18)
Household visits by HEWs	49	44	51	53	7 (3, 11)	9 (4, 14)
Household with Family Health Card	54	49	53	60	4 (−0, 9)	11 (6, 16)
Family planning						
Contraceptive prevalence Rate	48	44	48	52	4 (−1, 8)	7 (2, 13)
Maternal and newborn						
ANC 4+	51	44	54	55	10 (5, 15)	11 (5, 17)
Neonatal tetanus Protected childbirth	68	65	68	64	3 (−2, 7)	-1 (−6, 4)
Institutional deliveries	56	50	53	59	4 (−3, 11)	9 (1, 17)
Early PNC	9	8	9	9	1 (−1, 3)	1 (−1, 3)
Clean cord care	33	30	24	38	-6 (−12, 1)	8 (0, 16)
Thermal care	42	35	36	41	1 (−6, 8)	5 (−2, 13)
Immediate breastfeeding	70	62	63	65	1 (−6, 7)	3 (−5, 10)
Childhood immunization						
Fully vaccinated	65	62	66	65	4 (−1, 10)	3 (−4, 10)
Dropout between Penta 1 and Penta 3	11	13	10	12	-3 (−6, 0)	−2 (−6, 2)

*ANC + 4* Received four or more antenatal care visits, *HEP* Health Extension Program, *HEWs* Health extension workers, *PNC* Postnatal care, *WDA* Women’s Development Army

Density of WDAs, which can be viewed as penetration of the HEP, was also associated with three measures of uptake of services among women: contraceptive prevalence rate, four or more ANC visits and institutional delivery. Respectively, these were 7, 11 and 9 percentage-points higher (*p* < .05) among respondents from higher WDA density areas, compared with those from lower WDA density areas. Although clean cord care was also associated with WDA density, the association was not in the expected direction. We found no evidence that WDA strategy implementation strength was associated with childhood immunization, neonatal tetanus protected childbirth, thermal care, immediate breastfeeding or early postnatal care (PNC).

## Discussion

Many countries have produced and deployed community health workers as an alternative health workforce to overcome the critical shortage of health staff [14, 34]. The community health structure of Ethiopia comprises the HEP, with HEWs supported by the WDA [9, 23, 27].

The density of the WDA was associated with wealth quintile, suggesting that while WDA density is not as high as desired in all areas, the distribution of higher density WDA appears to be pro-poor. McColloum et al., in a recent systematic review reported that community health worker programs were equitable, including in terms of socio-economic position and geographical location, based on their review of 34 community health worker interventions from all regions of the world [35].

The findings of this study showed that, the contraceptive prevalence rate was positively associated with higher WDA strategy implementation strength, among this sample of mainly married women. The contraceptive prevalence rate among currently married women has increased in Ethiopia from 8 to 36% in the last sixteen years [7, 36], and the Health Sector Transformation Plan target set for the year 2020 is 55%. Our results suggest that the WDA strategy can help to reach that target.

In this study, four or more ANC visits had an overall prevalence of 51%, that was again higher in high WDA density areas. This overall prevalence is higher than the current national average (32%) [7]. Repeated ANC attendance is one of the most important indicators that shows the quality of health services, hence additional effort is required for this service to excel. Repeated ANC visits, at least four during pregnancy, provide opportunities for reaching pregnant women with a number of interventions at different stages of pregnancy [9, 37]. A Cochrane review of community-based services for maternal care from 2010, reported similar findings of increased access to ANC where community health workers were present, across a variety of settings [38].

Institutional delivery in this survey was 56% overall and more elevated in areas with higher WDA density. Overall, institutional delivery was higher in our study population when compared with the Ethiopia Demographic Health Survey (DHS) 2016 report, which showed 28% [7]. Currently, Ethiopia is experiencing an unprecedented acceleration in institutional delivery, following many years of little change despite being a priority focus for the Federal Ministry of Health. For instance, reports for three consecutive Ethiopia DHS, 2010, 2014 and 2016, showed a rise in institutional deliveries from 10, to 15%, to 28%, respectively. This increase is likely to be a multi-faceted process, including the government’s efforts and investments under a national movement with the logo “No mother should die while giving life”, amplified by community involvement through the WDA. In their systematic review, Gilmore and McAulifee suggest that increased contact points with community health workers may have an increased impact [21]. Increased density of the WDA may facilitate penetration of public health messages and work towards message saturation [39]. However, the performance is still far behind the Health Sector Transformation Plan institutional delivery target of 90% by the year 2020. For institutional delivery to be effective, it is essential to understand the factors that influence individuals and households to utilize institutions for delivery and develop effective interventions to promote respectful care in this context [40, 41].

The findings of this study showed immunization coverage is relatively high (65%), albeit far behind the Health Sector Transformation Plan target (95%). We found no evidence of association of the Expanded Program of Immunization with WDA density. This may be because the Expanded Program of Immunization started in Ethiopia over thirty years ago and is well established throughout the country. Client perceptions are shaped by cultural values, previous experiences, perceptions of the role of the health system and interactions with providers [42].

Interestingly, we found no evidence of an influence of WDA density on immediately initiating breastfeeding or thermal care (*p* > 0.05). Other community health worker programs have reported improvements in thermal care [43] and breastfeeding [44], and improving these essential life-saving interventions [9] requires more attention and further improvement.

Only 9% mothers in our study received early PNC, which is much lower than the 2016 Ethiopia DHS report (17%) [7]. Early PNC was low across all levels of WDA density. Given that the highest number of maternal deaths occur during labor, delivery and in the first few days after delivery, there is a critical need for good quality care during these periods [9, 45]. Different studies [46, 47] and consecutive Ethiopia DHS have also reported low PNC service coverage, suggesting the need for immediate action. Similar challenges in achieving adequate PNC coverage have been reported in other countries. For instance, a study analyzing data from 30 surveys conducted between 1999 and 2004, showed substantial increases worldwide in the use of ANC services, but wide gaps between ANC and delivery care and even more so between delivery care and PNC [37]. Recent data from Morocco (2016) [48], showed that the proportion of women who attended a postnatal consultation was only 30%. A meta-analysis conducted by Langlois et al. indicates that the use of PNC remains highly inequitable according to socioeconomic status, education and geographical access to health facilities [49]. There is a need to identify constraining factors on both the demand and supply sides and find appropriate solutions for improving coverage [41, 46, 47].

Analyzing the findings of our study across the continuum of care, the contraceptive prevalence rate, four or more ANC visits and skilled delivery were higher in areas with higher or moderate WDA density than in areas with low WDA density. Latrine coverage was also high in these areas, and improving hygiene and sanitation, including latrine coverage, plays a key role in the improvement of maternal and child health. The findings of this study showed that 54% had a Family Health Card, the communication tool used by HEWs to promote knowledge and behavior change. In line with this, Gilmore and McAuliffe’s systematic review also described the vital role of community health workers in facilitating the implementation of the continuum of care, acting as a bridge between the community and health facility [21].

The WDA strategy is an integral part of the HEP, thus WDA density can be seen as the penetration of the HEP. Consistent with the strategy, we found that kebeles with higher WDA implementation strength were associated with better outcomes. The WDA provides an effective platform to engage the community to bring significant practical and attitudinal change. As the HEP outreach activities reflect the performance of the HEWs, the observed effects of WDA density on the reproductive, maternal and newborn health indicators in this study are the combination of the performances of the HEWs and the active WDAs.

In sum, our study shows that strategies to train and deploy community health workers show great promise in increasing access to treatment and care of mothers and children.

### Limitations

There are several limitations to the study. First, due to the cross-sectional nature of the study, there remains temporal ambiguity between program exposure (WDA density) and the outcomes of interest. The WDA strategy implementation strength represented the situation of the WDA program during the three months before the survey. Thus, the assumption of the analysis is that the WDA strategy implementation strength remained the same when the outcomes of interest (for example, women seeking four or more ANC visits) took place. However, this may not have been the case. Second, the effect of WDA strategy implementation strength on the outcomes of interest may be biased due to confounding. Third, the generalizability of the study is limited to the 145 districts from which data were obtained. Fourth, the 30 by seven survey sampling method can be criticized because the interviewers may avoid hard-to-reach areas and non-responders are not revisited [31]. To minimize the bias the interviewers moved away from the center of the kebele towards the periphery to select subsequent households for interview; which was closely monitored by survey supervisors. Nonetheless, it is likely that some of the service utilization indicators could still be over-estimated. In addition, this study employed a quantitative survey method, hence, the data collected were not sufficient to make a detailed analysis to understand complex issues, for example the reasons for low PNC service uptake.

## Conclusion

Many studies conducted both nationwide and in selected regions of Ethiopia, have assessed the performance of HEWs and the WDA for improving RMNCH, yet the question of the density of active WDA leaders has not been addressed. This work analyzed the correlation of density (number) of active WDA leaders and uptake of RMNCH services in selected indicators. Most of the RMNCH indicators showed better results in areas with higher WDA strategy implementation strength areas than in areas with lower implementation strength. Several studies, including United Nations reports, have described how community health workers have contributed to improved access and uptake of health services, including RMNCH, in different contexts, especially for underserved populations in low and middle-income countries [14, 34].

The WDA initiative, through ensuring that every Ethiopian household is reached and involved in active participatory learning and sharing of experiences, aims to bring about transformational change in health behaviors and outcomes [23, 24]. Previous studies and official reports have shown that there have been major improvements in the health system since the introduction of the WDA implementation strategy [9, 23, 27, 47]. The present study also shows that a higher density of active WDA members has intensified the undertaking of HEWs to advance the implementation of RMNCH services, which is one of the four main components of HEP packages and a key priority of the country.

The lessons gained from this study may be important for other countries that have similar working setups. The need of further research on the effect of WDA density, such as, the challenges to achieving the ‘higher’ density of active WDA members, may help in accelerating WDA strategy implementation strength.

## Additional files

Additional file 1: Community-based strategies to improve maternal and newborn health in Ethiopia. Background to the Health Extension Program and the intervention strategies introduced under the L10 K project. (DOCX 58 kb)

Additional file 2: **Figure S1.** Intervention timeline. An implementation timeline for L10 K’s strategies. (PDF 50 kb)

Additional file 3: **Figure S2.** L10 K survey domain and strategy. A map showing woredas implementing the various program strategies. (PDF 541 kb)

Additional file 4: Kebele-level random effects logit model coefficients predicting the outcomes of interest and goodness-of-fit statistics. The coefficients of the multilevel models and their goodness-of-fit statistics (global Wald’s statistics). (DOCX 33 kb)

## Abbreviations

ANC: Antenatal care
DHS: Demographic Health Survey
HEP: Health Extension Program
HEW: Health Extension Worker
L10 K: Last 10 Kilometers Project
PNC: Postnatal Care
RMNCH: Reproductive Maternal Neonatal and Child Health
SNNP: Southern Nations, Nationalities and Peoples
WDA: Women’s Development Army
