# Supplementary material for: Correlates of the Women’s Development Army strategy implementation strength with household reproductive, maternal, newborn and child healthcare practices: a cross-sectional study in four regions of Ethiopia
Source: BMC Pregnancy Childbirth. 2018 Sep 24;18(Suppl 1):373. doi: 10.1186/s12884-018-1975-y (PMC6157249; doi:10.1186/s12884-018-1975-y)
Supplement: Supplementary file 1 — Community-based strategies to improve maternal and newborn health in Ethiopia. Background to the Health Extension Program and the intervention strategies introduced under the L10 K project. (DOCX 58 kb) [file 12884_2018_1975_MOESM1_ESM.docx]

# Additional file 1: Community-based strategies to improve maternal and newborn health in Ethiopia

## The Ethiopian Health System

Ethiopia has a three-tier health system. At the first level, the district (*woreda*) health system serves a population of about 100,000 people; at the second level, a general hospital serves about 10 woredas with a population of about one million people; and at the third level, a specialized hospital serves the catchment population of about five million people. The rural district primary health care system comprises three to four primary health care units supported by one primary or district hospital with comprehensive emergency obstetric and newborn care. Primary health care units are formed of one health center for every 25,000 people in the woreda, with five satellite health posts [1].

Health center staff include health officers, nurses, midwives and laboratory technicians, each with an undergraduate diploma or health degree. They provide preventive and curative services including basic emergency obstetric and newborn care (BEmONC), and supervise the satellite health posts. Ethiopia’s flagship Health Extension Program comprises the health posts, each with two female Health Extension Workers (HEWs) serving a community (*kebele*) of about 5,000 people with basic community-based promotive, preventive and selective curative health services [2]. The package of services provided by the Heath Extension Program is given in Table S1.

To further extend the reach of the Health Extension Program and mobilize the community and households, a network of Women’s Development Army (WDA) members, also known as Health Development Army, was established. A network was created between five households, based on social and geographical proximity, and one WDA member, to influence one another to adopt and practice healthy behaviors. For every six WDA members, and thus for every 30 households, one WDA member was chosen as a ‘1-to-30’ team leader [3].

**Table S1: Components of the Health Extension Program service package**

| Pillars of the Health Extension Program | Health Extension Program packages |
| --- | --- |
| Hygiene and Environmental Health services | Construction, maintenance and utilization of latrine |
|  | Food hygiene |
|  | Water storage and safety |
|  | Healthy housing |
|  | Solid and liquid waste management |
|  | Personal hygiene |
|  | Control of insects and rodents |
| Family Health | Family planning |
|  | Adolescent reproductive health |
|  | Maternal and child health |
|  | Expanded Program of Immunization |
|  | Nutrition |
| Disease Prevention and Control | HIV/AIDS and TB prevention and control |
|  | Malaria prevention and control |
|  | First Aid |
| Health Education and Communication | Health education and communication |

## The Last Ten Kilometers Project

The Last Ten Kilometers Project (L10K) supported the Health Extension Program in providing high impact maternal, newborn and child health services through innovative community-based strategies . L10K, in partnership with 12 local civil society organizations enhanced the interactions between frontline health workers and communities to achieve more accessible, efficient and equitable maternal, newborn and child health services [4]. The L10K intervention area covered 115 woredas in Amhara, Oromia, Southern Nations, Nationalities and Peoples’ (SNNP) and Tigray regions, with a population of 17 million people, approximately 19% of Ethiopia’s population. The project timeline was from October 2007 to September 2012, with an extension to September 2015 with a modified scope and strategies. The first paper in this supplement focusses on the Women’s Development Army, which was introduced by the government in 2011, while the remaining papers in the supplement focus on the effectiveness of the community engagement strategies implemented by L10K between December 2012 and December 2014. An implementation timeline for L10K’s strategies is given in Figure S1.

**Figure S1: Intervention timeline**

Participatory Community Quality Improvement (PCQI) was introduced in December 2012 in 16 primary health care units in 14 woredas; and the Early Care Seeking and Referral Solutions strategy was initiated in December 2012 in eight primary health care units in eight woredas, and then extended to eight further primary health care units in eight further woredas in December 2013.

PCQI ensured continuous quality improvement through a cyclical process that involved the identification of barriers to quality of services, developing an action plan to address those barriers, implementing the action plan, and assessing the improvement. Barriers to quality of services were identified through community discussion forums as well as through assessing of health posts. Thorough discussion, problems were identified, solutions were forwarded and action plans were developed. A quality improvement team led by the kebele manager and the HEWs regularly monitored implementation of the action plan and assessed improvements. The PCQI strategy was implemented in 16 primary health care units located in 14 woredas covering a population of about 424,000 people.

With Early Care Seeking and Referral Solutions, community representatives (WDA members, administrators and leaders) and health care providers from different levels of the health system, including hospitals providing comprehensive emergency obstetric care, health centers providing BEmONC, and health posts, came together for a two-day meeting every quarter to identify barriers and map community resources for critical maternal and newborn health services. This information was used in a participatory process, to prioritize identified barriers and develop solutions to address them. The primary health care unit and the woreda health office then took charge in actively managing the referral system between the different levels of health care. During the study period this intervention was implemented in 16 primary health care units located in 16 L10K woredas.

BEmONC services were strengthened in 2013 in 134 primary health care units, through training, mentorship and providing essential equipment and supplies, if needed. This BEmONC strengthening was conducted in all PCQI, Early Care Seeking and Referral Solutions areas.

Three community-based strategies—Community-Based Data for Decision-Making (CBDDM), Family Conversation and Birth Notification—were implemented in the 115 woredas that constituted the L10K Platform. CBDDM, introduced in July 2013, fostered the Health Extension Program to generate and use data for improving maternal and newborn health care practices. CBDDM aimed to improve the skills of HEWs in organizing the WDA network, enabled WDA ‘1-30’ team leaders to map their network of 25 to 30 households and to maintain surveillance, to maximize uptake of maternal and newborn health care services in households. CBDDM was used to identify pregnant women and ensure they received antenatal, intrapartum and postpartum care, and that their infants received postnatal care, essential newborn care, immunization and growth promotion services.

The Family Conversation strategy was introduced early in 2014 to promote birth preparedness and essential newborn care; and the Birth Notification strategy was introduced in mid-2014 to promote early postnatal care. In addition, L10K encouraged HEWs to work with kebele administrations to organize community festivals to give public recognition to the WDA members, both to motivate them and to sustain their engagement.

A map showing woredas implementing the various program strategies is shown in Figure S2 and Table S2 respectively. PCQI, BEmONC and Early Care Seeking and Referral Solutions were implemented in some of the L10K Platform. Nonetheless the distribution of kebeles, primary health care units and population in Table S2 was mutually exclusive.

**Figure S2: L10K survey domain and strategy**

**Table S2: Sample unit sizes by survey period and L10K program domains**

## Surveys to evaluate the effectiveness of community engagement strategies on maternal, newborn and child health care

Representative before-and-after household and health post surveys were conducted in December 2010-January 2011 and December 2014-January 2015 in the 115 L10K intervention woredas and in 30 comparison woredas. The comparison woredas were purposely selected from the same administrative zones and comprised six woredas from Tigray and eight woredas each from the other three regions.

The household surveys used two-stage stratified cluster sampling to obtain family planning related information from women of reproductive age (15 to 49 years); maternal, newborn and infant health and nutrition related measurements from women with children aged 0 to 11 months; child immunization and childhood illnesses related measurements from women with children aged 12 to 23 months; and Health Extension Program coverage, health systems support for the Health Extension Program, WDA strategy activities, and exposure to the CBDDM strategy, from HEW interviews and a health post record review. Four survey instruments were used, three women’s questionnaires and one health post questionnaire. The women’s questionnaires were pre-coded and translated into three major local languages, Amharic, Oromifa and Tigrigna. In SNNP, where there are at least 11 further local languages, local interviewers translated questions from Amharic informally.

The household survey instruments were similar for the two survey periods. However, items were added to the follow-up survey instruments to measure the community engagement strategies introduced after January 2011.

### *December 2010 – January 2011 Survey*

The sample size for the 2010-11 household survey was planned in order to detect improvements in maternal, newborn and child health behaviors and practices within each of the four regions and to detect the effects of community engagement strategies including PCQI and CBDDM. Robust sample sizes were estimated to detect difference between two surveys or two groups of an indicator that had the highest variances (i.e. expected prevalence of the two surveys or groups would have equal percentage-points differences from 50%). For all estimates the power was set at 80%, two-tailed alpha error at 0.05, design effect 2, and 12 respondents per cluster. Ballpark estimates of survey costs were also considered while estimating sample sizes. First, the sample size was estimated for a two-stage cluster survey to detect a change in an indicator of at least 9 percentage-points between baseline and follow-up surveys (from 45% to 54%). Then, the sample size for the PCQI area was increased to detect a difference of at least 12 percentage-points in an indicator between L10K intervention areas without and with the PCQI strategy (respectively assumed to be 44% and 56%). Lastly, the sample size for the comparison area was estimated to detect a difference of at least 8 percentage-points in an indicator between L10K and non-L10K intervention areas (assumed to be 46% and 54% respectively). The sample size estimation procedure was used for all three target groups of respondents of the household survey.

We had planned to compare areas with and without each specific strategy using a difference-in-differences approach. However, implementation priorities changed; for example, the Health Extension Program introduced the WDA strategy, and CBDDM, Family Conversation, Birth Notification, BEmONC and Early Care Seeking and Referral Solutions were implemented at different points in time during the study period, so the original before-and-after intervention-and-comparison design was only feasible for estimating the effectiveness of PCQI.

At the first stage of the cluster sampling, kebeles were selected as the primary sampling units with the probability proportional to their population size and stratified by administrative region and program strategy. At the second stage of the cluster sampling, the World Health Organization 30 by 7 cluster sample strategy [5] was used to obtain information from 12 respondents in each target population, from each kebele. The interviewers first went to the middle of the kebele and randomly selected a household, moving in a straight line from center of the kebele to its periphery, they then visited every fifth household and interviewed all the women in that household if they were within the target population. If a woman aged 15-49 had a child between 0 to 11 months of age she was interviewed for the women of reproductive age questionnaire as well as for the questionnaire for women with children aged 0 to 11 months. After completion of the quota for women of reproductive age in a kebele the interviewers then focused on completing interviews for the other target groups.

One health post questionnaire per kebele was completed by interviewing the HEWs and reviewing the health post documents. If more than one HEW was present, they were interviewed together, after seeking their written consent. In the few cases where there was more than one health post in a kebele, one health post was randomly selected for the HEW interview and households for interview were selected from within the catchment area of that health post.

The fieldwork was carried out by 32 teams, with each team comprising one supervisor and four interviewers. Overall, in the four regions 128 interviewers, 32 supervisors and 10 regional survey coordinators were deployed. The interviewers were health professionals working for regional health bureaus at zonal or woreda level and were recruited in consultation with the regional health bureaus. The supervisors were mostly from L10K’s implementing partners. To avoid bias, supervisors and interviewers did not conduct survey work in their own areas. In addition, six consultants trained and supervised the survey teams.

Field staff were trained in three sessions: Amhara and SNNP region field teams were trained together in Addis Ababa; the Oromia region team was trained in Jimma; and the team from Tigray region was trained in Mekelle. Each session lasted five days and included a general introduction about the concepts and objectives of L10K, classroom instruction on interview techniques and field survey procedures, a detailed review of each item in the questionnaires, specific survey instructions and role play. A one-day field practice was also part of the training. Survey supervisors and regional survey coordinators were orientated on how to organize the survey, monitor and supervise the field work, and on techniques for detecting and correcting data errors in the field. Training and data collection were completed in six weeks from December 2010 to January 2011.

A total of 327 kebeles from 115 L10K intervention woredas and 79 kebeles from 30 comparison area woredas were visited, in which 12,275 women were interviewed, including 4,872 women of reproductive age, 4,871 women with children aged 0 to 11 months, and 4,860 women with children aged 12 to 23 months (Table S2).

### *December 2014 – January 2014 Survey*

The 2014-15 survey revisited 402 of the 406 kebeles sampled during 2010-11 survey and oversampled Early Care Seeking and Referral Solutions, and PCQI areas. The sample for women of reproductive age was also increased in the areas where L10K was planning to implement new family planning interventions in the future. Considering internal comparison group design, robust sample sizes were estimated (as described before) to detect 12, 8 and 10 percentage-point effects of Early Care Seeking and Referral Solutions, PCQI and family planning respectively. Over sampling was done by randomly selecting a larger number of kebeles from those areas. Table S2 shows the distribution of the 2014-15 survey sampling units according to the L10K program strategy domains.

## *Data quality assurance*

The data collectors were not professional interviewers but regional health bureau staff who were eager to visit households and learn from their target population. Training was one of the essential steps for data quality. The 2010-11 survey data were collected using paper questionnaires; while the 2014-15 survey data were collected and archived using a web based mHealth platform, SurveyCTO, using smart phones [6]. After completing the interviews, the team supervisors reviewed the responses for completeness before leaving the kebele and the respondents were revisited if required. The regional survey coordinators randomly visited the survey teams and a small number of households to validate responses.

Epi Info 6 was used for data entry and management of the 2010-11 survey data, allowing only valid responses and with skip patterns to minimize data entry error. Data were entered twice by different data entry clerks and the differences corrected by revisiting the responses in the hard copies of the questionnaires.

## Overview of study designs for the papers in this supplement

***Correlates of the Women’s Development Army strategy implementation strength with household reproductive, maternal, newborn and child health care practices ― a cross-sectional study in four regions of Ethiopia****.* This used the 2014-15 survey data to assess the association between WDA team leader density of a kebele and household maternal, newborn and child health care behaviors and practices. This study did not use the 2010-11 survey as a baseline because of the presence of community health promoters, a precursor to the WDA [7].

***Effects of a Community-Based Data for Decision-Making intervention on health care practices in Ethiopia ― a dose-response study.*** This used the data from the 195 kebeles where only the L10K platform activities were being implemented and which were visited during both surveys. The study assessed whether kebele-by-kebele changes in implementation strength of the CBDDM strategy were associated with improvements in maternal, newborn and child health care behaviors and practices, controlling for exposure to self-reported Community Conversation.

***Effects of a Participatory Community Quality Improvement strategy on improving household and provider health care behaviors and practices ― a propensity score analysis.***

This used data from 116 kebeles which were visited during both 2010-11 and 2014-15 to assess whether PCQI in addition to CBDDM and BEmONC (34 kebeles) was more effective in improving maternal and newborn health care practices than CBDDM and BEmONC alone (82 kebeles).

***Effects of Family Conversation on maternal and newborn health care practices in Ethiopia ― a propensity score- matched analysis.*** This used the 2014-15 survey data to assess whether self-reported exposure to Family Conversation was associated with more institutional deliveries, more postnatal care and improved essential newborn care practices.

## ABBREVIATIONS

| BEmONC | Basic emergency obstetric and newborn care |
| --- | --- |
| CBDDM | Community-based data for decision-making |
| HEW | Health Extension Worker |
| L10K | Last 10 Kilometers |
| PCQI | Participatory Community Quality Improvement |
| SNNP | Southern Nations, Nationalities and Peoples |
| WDA | Women’s Development Army |

**ADDITIONAL FILES**

**File name:** Paper 1_Additional file 2_Figure S1

**File format:** .pdf

# Title of data: Figure S1: Intervention timeline

**Description of data:** An implementation timeline for L10K’s strategies

**File name:** Paper 1_Additional file 3_Figure S2

**File format:** .pdf

# Title of data: Figure S2: L10K survey domain and strategy

**Description of data:** A map showing woredas implementing the various program strategies

## REFERENCES

1. Federal Democratic Republic of Ethiopia Ministry of Health (FMOH). Health Sector Development Program IV: 2010/11–2014/15. Addis Ababa: FMOH; 2010. <https://phe-ethiopia.org/admin/uploads/attachment-721-HSDP%20IV%20Final%20Draft%2011Octoberr%202010.pdf>. Accessed 15 Apr 2018.

2. Wakabi W. Extension workers drive Ethiopia’s primary health care. Lancet. 2008; doi:10.1016/S0140-6736(08)61381-1.

3. Admasu K, Balcha T, Getahun H. Model villages: A platform for community-based primary health care. Lancet. 2016; doi: 10.1016/S2214-109X(15)00301-0.

4. Darmstadt GL, Marchant T, Claeson M, Brown W, Morris S, Donnay F, et al. A strategy for reducing maternal and newborn deaths by 2015 and beyond. BMC Pregnancy Childbirth. 2013;13:216.

5. Lemeshow S, Robinson D. Surveys to measure programme coverage and impact: A review of the methodology used by the expanded programme on immunization. World Heal Stat Q. 1985;38:115–32.

6. Dobility I. SurveyCTO. http://www.surveycto.com. Accessed 3 May 2017.

7. Karim AM, Admassu K, Schellenberg J, Alemu H, Getachew N, Ameha A, et al. Effect of Ethiopia’s Health Extension Program on maternal and newborn health care practices in 101 rural districts: A dose-response study. PLoS One. 2013;8(6):e65160.

8. Stuart EA, Rubin DB. Best practices in quasi experimental designs: Matching methods for causal inference. In: Osborne J, editor. Best practices in quantitative methods. Thousand Oaks, California: Sage Publications, Inc.; 2008. p. 155–76.

9. StataCorp. Stata Treatment-Effects Reference Manual: Potential Outcomes/Counterfactual Outcomes, Release 14. College Station, TX: StataCorp LP; 2015.

10. Rosenbaum P, Rubin D. Constructing a control group using multivariate matched sampling methods that incorporate the propensity score. Am Stat. 1985;28:3083–107.

11. Austin PC. Balance diagnostics for comparing the distribution of baseline covariates between treatment groups in propensity-score matched samples. Stat Med. 2009;28:3083–107.

| **Table S2: Sample unit sizes by survey period and L10K program domains** | | | | | | | | | | | | | | | | |
| --- | --- | --- | --- | --- | --- | --- | --- | --- | --- | --- | --- | --- | --- | --- | --- | --- |
| Program domain | Administrative units and population | | | |  | December 2010-January 2011 survey sample units | | | | |  | December 2014-January 2015 survey sample units | | | | |
|  | # of kebeles | # of primary health care units | # of *woredas | Population size (2007 census) |  | No of respondents | Women 15-49 | Women with children 0-11 months | Women with children 12-23 months | # of kebeles |  | No of respondents | Women 15-49 | Women with children 0-11 months | Women with children 12-23 months | # of kebeles |
|  |  |  |  |  |  | Full sample | | | | | | | | | | |
| L10K area | 3,070 | 579 | 115 | 14,405,559 |  | 9,880 | 3,924 | 3,923 | 3,912 | 327 |  | 11,820 | 5,170 | 4,801 | 4,822 | 400 |
| Platform (CBDDM, Family Conversation & Birth Notification) | 2,150 | 445 | 59 | 10,223,614 |  | 5,985 | 2,352 | 2,352 | 2,352 | 196 |  | 6,064 | 2,618 | 2,469 | 2,473 | 206 |
| Early Care Seeking and Referral Solutions (x) | 106 | 16 | 16 | 507,046 |  | 416 | 168 | 168 | 168 | 14 |  | 1,214 | 624 | 476 | 475 | 40 |
| PCQI (x) | 93 | 16 | 14 | 424,294 |  | 1,025 | 408 | 408 | 408 | 34 |  | 2,005 | 838 | 818 | 835 | 68 |
| BEmONC | 721 | 102 | 61 | 3,250,605 |  | 2,454 | 996 | 995 | 984 | 83 |  | 2,537 | 1,090 | 1,038 | 1,039 | 86 |
| Comparison | 740 | NA | 30 | 3,088,322 |  | 2,395 | 948 | 948 | 948 | 79 |  | 2,229 | 936 | 939 | 936 | 78 |
| Total | 3,810 | NA | 145 | 17,493,881 |  | 12,275 | 4,872 | 4,871 | 4,860 | 406 |  | 14,049 | 6,106 | 5,740 | 5,758 | 478 |
|  |  |  |  |  |  | Sample from kebeles that were visited during both the survey periods | | | | | | | | | | |
| L10K area |  |  |  |  |  | 9,781 | 3,888 | 3,887 | 3,876 | 324 |  | 9,449 | 3,988 | 3,883 | 3,891 | 324 |
| Platform (CBDDM, Family Conversation & Birth Notification) |  |  |  |  |  | 5,954 | 2,340 | 2,340 | 2,340 | 195 |  | 5,696 | 2,398 | 2,338 | 2,341 | 195 |
| Early Care Seeking and Referral Solutions (x) |  |  |  |  |  | 383 | 156 | 156 | 156 | 13 |  | 347 | 161 | 147 | 152 | 13 |
| PCQI (x) |  |  |  |  |  | 1,025 | 408 | 408 | 408 | 34 |  | 1,006 | 421 | 408 | 420 | 34 |
| BEmONC |  |  |  |  |  | 2,419 | 984 | 983 | 972 | 82 |  | 2,400 | 1,008 | 990 | 978 | 82 |
| Comparison |  |  |  |  |  | 2,365 | 936 | 936 | 936 | 78 |  | 2,229 | 936 | 939 | 936 | 78 |
| Total |  |  |  |  |  | 12,146 | 4,824 | 4,823 | 4,812 | 402 |  | 11,678 | 4,924 | 4,822 | 4,827 | 402 |
| NA: data not available. SA  *Program domain within a woreda may overlap. The PCQI, Early Care Seeking and Referral Solutions, and BEmONC woredas also include kebeles which had only been exposed to the Platform strategy. As such, distribution of woredas by L10K program domain is more than 115.  (x) PCQI and Early Care Seeking and Referral Solutions primary health care units also included the BEmONC and the Platform strategies. | | | | | | | | | | | | | | | | |
