# Supplementary material for: Correlates of the Women’s Development Army strategy implementation strength with household reproductive, maternal, newborn and child healthcare practices: a cross-sectional study in four regions of Ethiopia
Source: BMC Pregnancy Childbirth. 2018 Sep 24;18(Suppl 1):373. doi: 10.1186/s12884-018-1975-y (PMC6157249; doi:10.1186/s12884-018-1975-y)
Supplement: Supplementary file 2 — Figure S1. Intervention timeline. An implementation timeline for L10 K’s strategies. (PDF 50 kb) [file 12884_2018_1975_MOESM2_ESM.pdf]

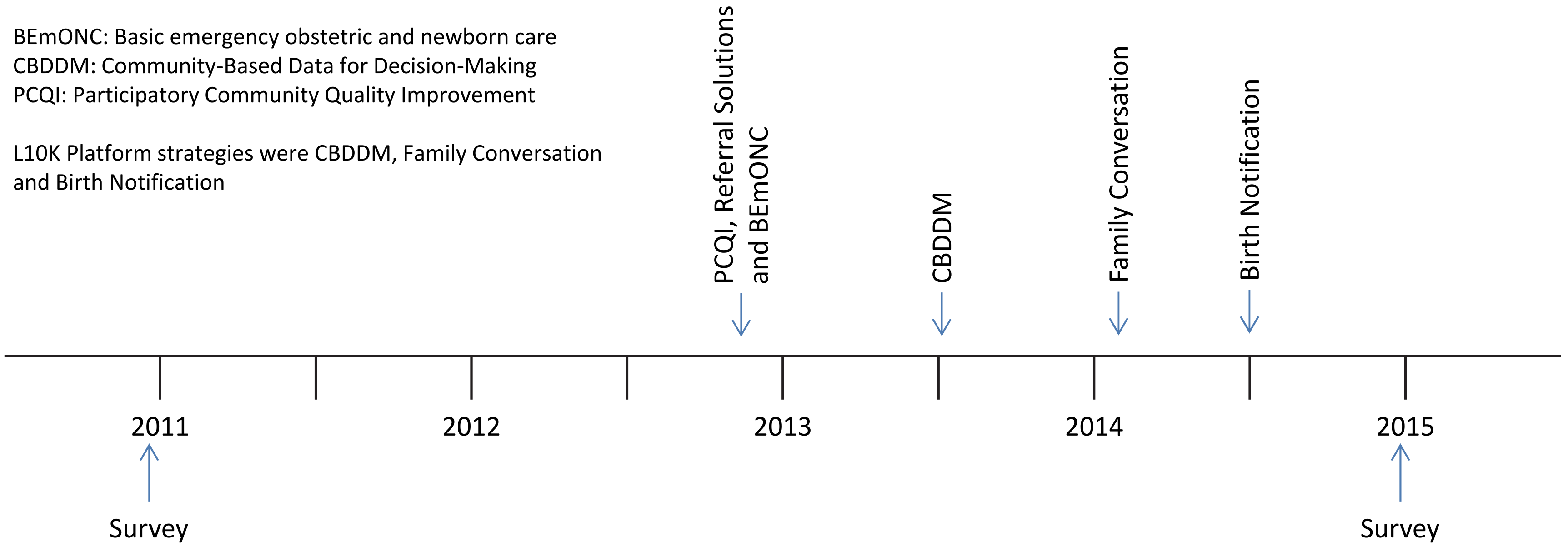
BEmONC: Basic emergency obstetric and newborn care
CBDDM: Community-Based Data for Decision-Making
PCQI: Participatory Community Quality Improvement
L10K Platform strategies were CBDDM, Family Conversation and Birth Notification
PCQI, Referral Solutions and BEmONC
CBDDM
Family Conversation
Birth Notification
2011
2012
2013
2014
2015
Survey
Survey
