# Supplementary figures and images for: Correlates of the Women’s Development Army strategy implementation strength with household reproductive, maternal, newborn and child healthcare practices: a cross-sectional study in four regions of Ethiopia
Source: BMC Pregnancy Childbirth. 2018 Sep 24;18(Suppl 1):373. doi: 10.1186/s12884-018-1975-y (PMC6157249; doi:10.1186/s12884-018-1975-y)

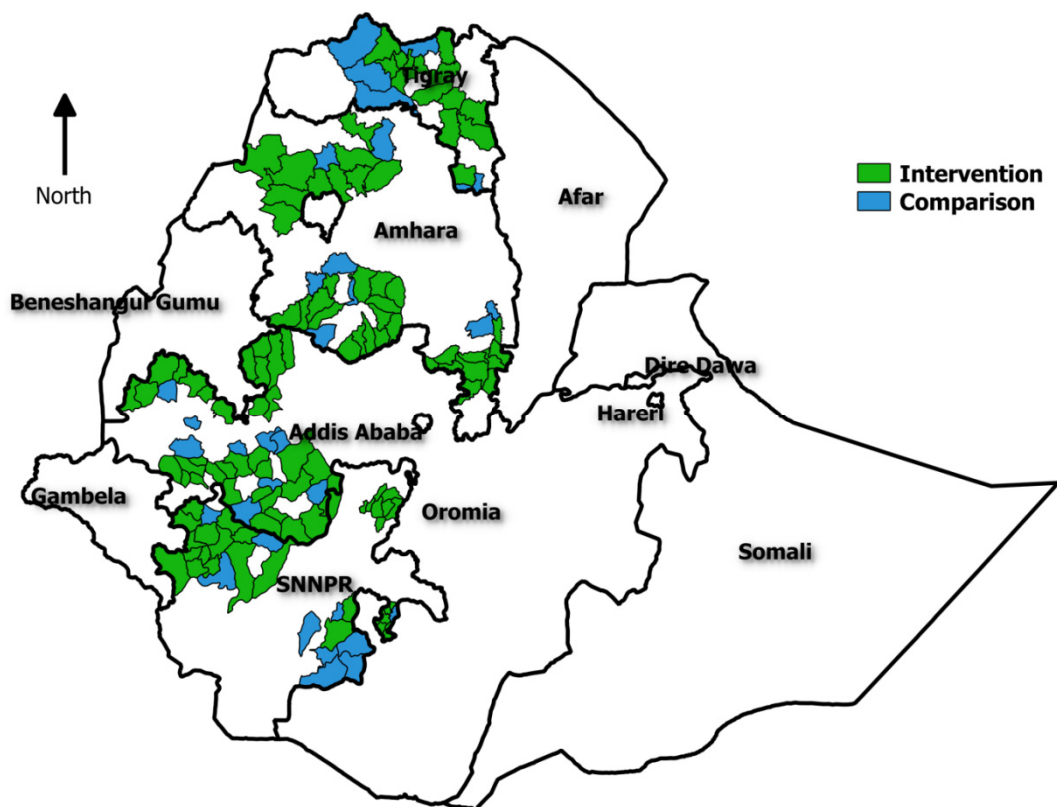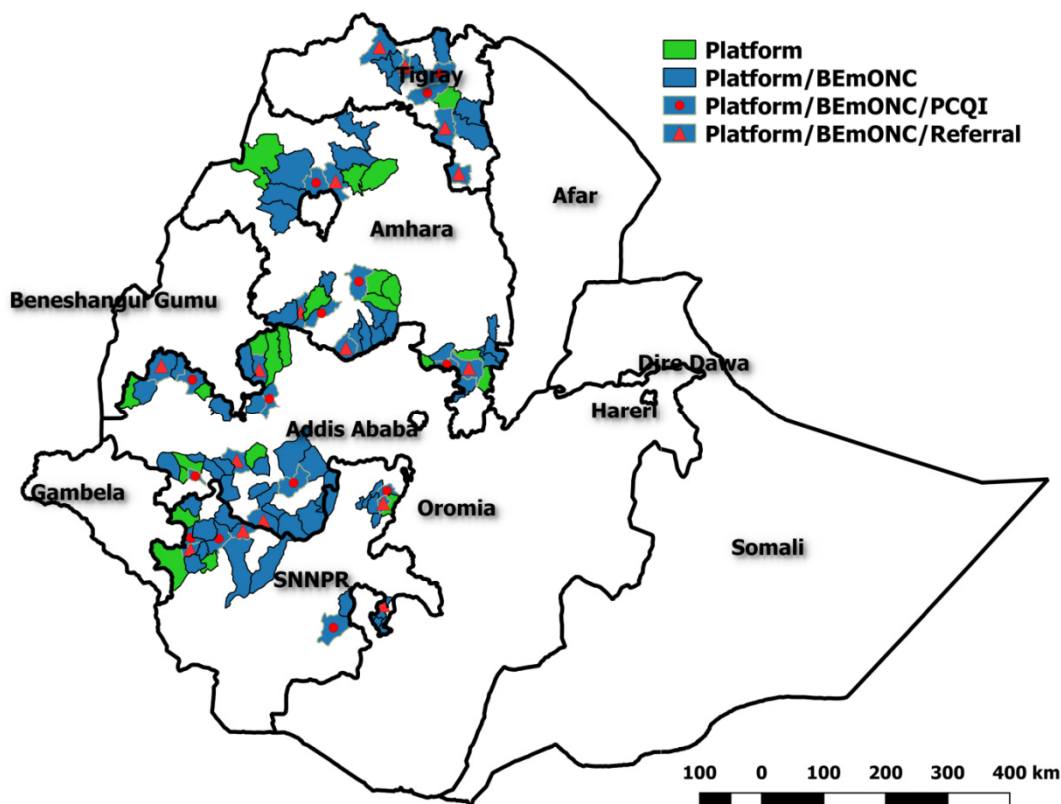

Supplement: Supplementary file 3 — Figure S2. L10 K survey domain and strategy. A map showing woredas implementing the various program strategies. (PDF 541 kb) [file 12884_2018_1975_MOESM3_ESM.pdf]
