# Supplementary material for: Correlates of the Women’s Development Army strategy implementation strength with household reproductive, maternal, newborn and child healthcare practices: a cross-sectional study in four regions of Ethiopia
Source: BMC Pregnancy Childbirth. 2018 Sep 24;18(Suppl 1):373. doi: 10.1186/s12884-018-1975-y (PMC6157249; doi:10.1186/s12884-018-1975-y)
Supplement: Supplementary file 4 — Kebele-level random effects logit model coefficients predicting the outcomes of interest and goodness-of-fit statistics. The coefficients of the multilevel models and their goodness-of-fit statistics (global Wald’s statistics). (DOCX 33 kb) [file 12884_2018_1975_MOESM4_ESM.docx]

| Kebele-level random effects logit model coefficients predicting the outcomes of interest and goodness-of-fit statistics | | | | | | | | | | | | | | | | |
| --- | --- | --- | --- | --- | --- | --- | --- | --- | --- | --- | --- | --- | --- | --- | --- | --- |
| Independent variables |  | Households with latrine  (N=12,381) | | | Household visits by HEWs  (N=12,381) | | | Household with Family Health Card  (N=12,381) | | | Contraceptive prevalence rate (N=4,777) | | | ANC 4+  (N=5,080) | | |
|  |  | Coef. | SE | p-value | Coef. | SE | p-value | Coef. | SE | p-value | Coef. | SE | p-value | Coef. | SE | p-value |
| Age |  | 0.015 | 0.006 | 0.013 | <.001 | 0.005 | 0.934 | -0.014 | 0.005 | 0.004 | -0.026 | 0.007 | <.001 | 0.023 | 0.008 | 0.005 |
| Education |  | 0.029 | 0.010 | 0.004 | 0.033 | 0.007 | <.001 | 0.063 | 0.007 | <.001 | 0.042 | 0.012 | <.001 | 0.066 | 0.011 | <.001 |
| Marital status | Other |  |  |  |  |  |  |  |  |  |  |  |  |  |  |  |
|  | In union | 0.139 | 0.116 | 0.233 | -0.038 | 0.094 | 0.684 | 0.174 | 0.098 | 0.075 |  |  |  | 0.542 | 0.188 | 0.004 |
| Number of | 0 |  |  |  |  |  |  |  |  |  |  |  |  |  |  |  |
| Children | 1 | -0.289 | 0.160 | 0.071 | 0.802 | 0.122 | <.001 | 1.269 | 0.128 | <.001 | 1.089 | 0.184 | <.001 |  |  |  |
|  | 2 | -0.225 | 0.171 | 0.188 | 0.971 | 0.131 | <.001 | 1.686 | 0.137 | <.001 | 1.465 | 0.195 | <.001 | -0.005 | 0.104 | 0.963 |
|  | 3 | -0.208 | 0.179 | 0.246 | 0.957 | 0.137 | <.001 | 1.897 | 0.144 | <.001 | 1.196 | 0.199 | <.001 | 0.052 | 0.118 | 0.658 |
|  | 4+ | -0.206 | 0.186 | 0.268 | 1.241 | 0.141 | <.001 | 1.981 | 0.149 | <.001 | 1.260 | 0.203 | <.001 | -0.164 | 0.128 | 0.200 |
| Religion | Orthodox |  |  |  |  |  |  |  |  |  |  |  |  |  |  |  |
|  | Protestant | 0.114 | 0.150 | 0.448 | -0.280 | 0.089 | 0.002 | 0.008 | 0.092 | 0.933 | -0.107 | 0.132 | 0.420 | 0.003 | 0.135 | 0.981 |
|  | Muslim | -0.021 | 0.168 | 0.902 | 0.089 | 0.102 | 0.387 | -0.088 | 0.108 | 0.417 | -0.454 | 0.138 | 0.001 | 0.059 | 0.145 | 0.683 |
|  | Other | -0.291 | 0.434 | 0.502 | -0.258 | 0.268 | 0.335 | 0.162 | 0.262 | 0.537 | -0.363 | 0.453 | 0.424 | 0.114 | 0.366 | 0.756 |
| Distance to any | <30 minutes |  |  |  |  |  |  |  |  |  |  |  |  |  |  |  |
| health facility | 30 min - <1 hr | -0.213 | 0.065 | 0.001 | -0.445 | 0.049 | <.001 | -0.408 | 0.050 | <.001 | -0.134 | 0.079 | 0.088 | -0.092 | 0.076 | 0.224 |
|  | 1+ hours | -0.353 | 0.084 | <.001 | -0.374 | 0.066 | <.001 | -0.230 | 0.067 | 0.001 | -0.258 | 0.109 | 0.018 | -0.287 | 0.098 | 0.003 |
| Wealth quintile | Lowest |  |  |  |  |  |  |  |  |  |  |  |  |  |  |  |
|  | Second | 0.840 | 0.072 | <.001 | 0.132 | 0.064 | 0.040 | 0.251 | 0.065 | <.001 | 0.036 | 0.104 | 0.725 | 0.103 | 0.100 | 0.301 |
|  | Middle | 1.125 | 0.082 | <.001 | 0.466 | 0.069 | <.001 | 0.582 | 0.071 | <.001 | 0.275 | 0.108 | 0.011 | 0.213 | 0.106 | 0.044 |
|  | Fourth | 1.811 | 0.100 | <.001 | 0.684 | 0.074 | <.001 | 0.682 | 0.076 | <.001 | 0.489 | 0.115 | <.001 | 0.320 | 0.112 | 0.004 |
|  | Highest | 2.770 | 0.130 | <.001 | 0.855 | 0.081 | <.001 | 0.849 | 0.083 | <.001 | 0.528 | 0.122 | <.001 | 0.600 | 0.121 | <.001 |
| WDA density | Lower |  |  |  |  |  |  |  |  |  |  |  |  |  |  |  |
|  | Moderate | 0.884 | 0.162 | <.001 | 0.310 | 0.097 | 0.001 | 0.183 | 0.105 | 0.080 | 0.164 | 0.100 | 0.102 | 0.427 | 0.110 | <.001 |
|  | Higher | 1.058 | 0.185 | <.001 | 0.401 | 0.112 | <.001 | 0.497 | 0.122 | <.001 | 0.316 | 0.118 | 0.007 | 0.473 | 0.127 | <.001 |
| HEW density | 2499 |  |  |  |  |  |  |  |  |  |  |  |  |  |  |  |
|  | 2,500 to 3,499 | -0.143 | 0.174 | 0.413 | 0.170 | 0.105 | 0.105 | -0.265 | 0.114 | 0.020 | 0.002 | 0.111 | 0.986 | 0.092 | 0.121 | 0.447 |
|  | 3,500 to 4,999 | 0.295 | 0.212 | 0.163 | -0.353 | 0.125 | 0.005 | -0.280 | 0.135 | 0.039 | -0.333 | 0.132 | 0.012 | -0.090 | 0.141 | 0.525 |
|  | 5,000+ | -0.201 | 0.237 | 0.396 | -0.755 | 0.146 | <.001 | -0.645 | 0.155 | <.001 | -0.390 | 0.151 | 0.010 | -0.062 | 0.162 | 0.700 |
| L10K area | No |  |  |  |  |  |  |  |  |  |  |  |  |  |  |  |
|  | Yes | -0.193 | 0.189 | 0.308 | 0.318 | 0.114 | 0.005 | 1.230 | 0.127 | <.001 | 0.410 | 0.121 | 0.001 | 0.197 | 0.128 | 0.125 |
| Region | Tigray |  |  |  |  |  |  |  |  |  |  |  |  |  |  |  |
|  | Amhara | 0.899 | 0.202 | <.001 | 0.127 | 0.128 | 0.319 | -0.360 | 0.138 | 0.009 | 0.498 | 0.138 | <.001 | -0.225 | 0.145 | 0.120 |
|  | Oromia | 1.801 | 0.259 | <.001 | -0.355 | 0.159 | 0.026 | -1.202 | 0.173 | <.001 | 0.619 | 0.186 | 0.001 | -0.273 | 0.197 | 0.164 |
|  | SNNP | 2.596 | 0.247 | <.001 | -0.645 | 0.148 | <.001 | -0.632 | 0.159 | <.001 | 0.474 | 0.168 | 0.005 | 0.244 | 0.179 | 0.173 |
| Constant |  | -1.647 | 0.328 | <.001 | -1.477 | 0.221 | <.001 | -2.230 | 0.236 | <.001 | -1.227 | 0.288 | <.001 | -1.305 | 0.322 | <.001 |
|  | | Statistics | | p-value | Statistics | | p-value | Statistics | | p-value | Statistics | | p-value | Statistics | | p-value |
| Global Wald’s chi-square (d.f.) | | 909 (25) | | <.001 | 529 (25) | | <.001 | 660 (25) | | <.001 | 234 (24) | | <.001 | 160 (24) | | <.001 |
| Random effects (d.f.) | | 1146 (1) | | <.001 | 649 (1) | | <.001 | 734 (1) | | <.001 | 82 (1) | | <.001 | 162 (1) | | <.001 |
| ANC 4+ = received four or more antenatal care visits; Coef. = Coefficient; HEW = Health extension worker; L10K = Last Ten Kilometers Project; SE = Standard error; SNNP = Southern Nations, Nationalities and Peoples; WDA = Women’s Development Army | | | | | | | | | | | | | | | | |

| Kebele-level random effects logit model coefficients predicting the outcomes of interest and goodness-of-fit statistics, continued … | | | | | | | | | | | | | | | | |
| --- | --- | --- | --- | --- | --- | --- | --- | --- | --- | --- | --- | --- | --- | --- | --- | --- |
| Independent variables |  | Neonatal tetanus protected childbirth  (N=5,080) | | | Institutional deliveries  (N=5,080) | | | Early PNC  (N=5,080) | | | Clean cord care  (N=2,309) | | | Thermal care  (N=2,309) | | |
|  |  | Coef. | SE | p-value | Coef. | SE | p-value | Coef. | SE | p-value | Coef. | SE | p-value | Coef. | SE | p-value |
| Age |  | 0.008 | 0.009 | 0.364 | 0.007 | 0.010 | 0.481 | 0.035 | 0.013 | 0.006 | -0.020 | 0.013 | 0.134 | 0.015 | 0.012 | 0.223 |
| Education |  | 0.062 | 0.012 | <.001 | 0.108 | 0.013 | <.001 | 0.057 | 0.017 | 0.001 | 0.064 | 0.020 | 0.001 | 0.051 | 0.020 | 0.010 |
| Marital status | Other |  |  |  |  |  |  |  |  |  |  |  |  |  |  |  |
|  | In union | 0.913 | 0.189 | <.001 | 0.153 | 0.224 | 0.496 | 0.807 | 0.384 | 0.036 | -0.030 | 0.333 | 0.929 | 0.320 | 0.335 | 0.339 |
| Number of | 1 |  |  |  |  |  |  |  |  |  |  |  |  |  |  |  |
| Children | 2 | 0.176 | 0.109 | 0.106 | -0.827 | 0.123 | <.001 | 0.336 | 0.168 | 0.045 | 0.046 | 0.176 | 0.796 | 0.275 | 0.171 | 0.107 |
|  | 3 | 0.118 | 0.123 | 0.339 | -0.834 | 0.138 | <.001 | 0.215 | 0.194 | 0.268 | 0.183 | 0.196 | 0.350 | 0.199 | 0.188 | 0.290 |
|  | 4+ | 0.248 | 0.134 | 0.064 | -0.906 | 0.150 | <.001 | 0.173 | 0.209 | 0.409 | 0.252 | 0.212 | 0.236 | -0.072 | 0.204 | 0.724 |
| Religion | Orthodox |  |  |  |  |  |  |  |  |  |  |  |  |  |  |  |
|  | Protestant | 0.075 | 0.138 | 0.587 | 0.044 | 0.164 | 0.788 | -0.637 | 0.240 | 0.008 | 0.328 | 0.222 | 0.140 | 0.192 | 0.209 | 0.359 |
|  | Muslim | 0.543 | 0.153 | <.001 | 0.070 | 0.189 | 0.712 | 0.298 | 0.210 | 0.155 | -0.133 | 0.240 | 0.578 | -0.204 | 0.223 | 0.361 |
|  | Other | -0.517 | 0.369 | 0.162 | -0.263 | 0.505 | 0.602 | -1.882 | 1.443 | 0.192 | 0.302 | 0.500 | 0.545 | 0.067 | 0.508 | 0.896 |
| Distance to any | <30 minutes |  |  |  |  |  |  |  |  |  |  |  |  |  |  |  |
| health facility | 30 min - <1 hr | -0.175 | 0.080 | 0.028 | -0.294 | 0.089 | 0.001 | -0.543 | 0.123 | <.001 | -0.150 | 0.123 | 0.221 | -0.301 | 0.117 | 0.010 |
|  | 1+ hours | -0.159 | 0.102 | 0.120 | -0.306 | 0.117 | 0.009 | -0.433 | 0.162 | 0.008 | -0.090 | 0.154 | 0.556 | -0.174 | 0.146 | 0.234 |
| Wealth quintile | Lowest |  |  |  |  |  |  |  |  |  |  |  |  |  |  |  |
|  | Second | -0.012 | 0.104 | 0.911 | 0.234 | 0.116 | 0.043 | -0.115 | 0.154 | 0.454 | 0.461 | 0.157 | 0.003 | 0.084 | 0.148 | 0.573 |
|  | Middle | -0.078 | 0.110 | 0.477 | 0.193 | 0.125 | 0.122 | -0.010 | 0.163 | 0.949 | 0.556 | 0.165 | 0.001 | 0.386 | 0.156 | 0.014 |
|  | Fourth | -0.024 | 0.117 | 0.841 | 0.520 | 0.134 | <.001 | -0.086 | 0.172 | 0.617 | 0.330 | 0.182 | 0.070 | 0.564 | 0.171 | 0.001 |
|  | Highest | 0.248 | 0.128 | 0.052 | 0.718 | 0.145 | <.001 | -0.192 | 0.183 | 0.293 | 0.446 | 0.202 | 0.027 | 0.121 | 0.194 | 0.533 |
| WDA density | Lower |  |  |  |  |  |  |  |  |  |  |  |  |  |  |  |
|  | Moderate | 0.129 | 0.113 | 0.251 | 0.185 | 0.178 | 0.296 | 0.086 | 0.145 | 0.552 | -0.297 | 0.172 | 0.084 | 0.053 | 0.160 | 0.743 |
|  | Higher | -0.040 | 0.128 | 0.754 | 0.449 | 0.206 | 0.029 | 0.131 | 0.163 | 0.422 | 0.392 | 0.195 | 0.045 | 0.252 | 0.184 | 0.171 |
| HEW density | 2499 |  |  |  |  |  |  |  |  |  |  |  |  |  |  |  |
|  | 2,500 to 3,499 | 0.077 | 0.123 | 0.531 | 0.105 | 0.192 | 0.586 | 0.164 | 0.150 | 0.275 | 0.039 | 0.188 | 0.834 | -0.314 | 0.174 | 0.071 |
|  | 3,500 to 4,999 | 0.110 | 0.146 | 0.451 | -0.390 | 0.230 | 0.090 | -0.005 | 0.184 | 0.980 | 0.357 | 0.213 | 0.093 | -0.472 | 0.202 | 0.020 |
|  | 5,000+ | -0.095 | 0.165 | 0.565 | -0.325 | 0.267 | 0.223 | -0.469 | 0.227 | 0.039 | -0.353 | 0.257 | 0.170 | -0.889 | 0.244 | <.001 |
| L10K area | No |  |  |  |  |  |  |  |  |  |  |  |  |  |  |  |
|  | Yes | 0.405 | 0.129 | 0.002 | 0.579 | 0.209 | 0.006 | 0.453 | 0.179 | 0.011 | 0.677 | 0.200 | 0.001 | 0.408 | 0.181 | 0.024 |
| Region | Tigray |  |  |  |  |  |  |  |  |  |  |  |  |  |  |  |
|  | Amhara | -0.805 | 0.148 | <.001 | -1.994 | 0.242 | <.001 | -0.016 | 0.175 | 0.925 | -0.318 | 0.245 | 0.194 | -0.691 | 0.229 | 0.002 |
|  | Oromia | -0.123 | 0.204 | 0.548 | -1.669 | 0.299 | <.001 | -0.408 | 0.268 | 0.128 | -0.249 | 0.335 | 0.458 | -0.661 | 0.312 | 0.034 |
|  | SNNP | -0.466 | 0.184 | 0.011 | -1.843 | 0.278 | <.001 | -0.647 | 0.246 | 0.008 | -0.562 | 0.303 | 0.064 | -1.238 | 0.282 | <.001 |
| Constant |  | -0.818 | 0.329 | 0.013 | 1.409 | 0.426 | 0.001 | -4.190 | 0.545 | <.001 | -0.761 | 0.527 | 0.149 | -0.628 | 0.507 | 0.215 |
|  | | Statistics | | p-value | Statistics | | p-value | Statistics | | p-value | Statistics | | p-value | Statistics | | p-value |
| Global Wald’s chi-square (d.f.) | | 194 (24) | | <.001 | 375 (24) | | <.001 | 110 (24) | | <.001 | 71 (24) | | <.001 | 93 (24) | | <.001 |
| Random effects | | 142 (1) | | <.001 | 602 (1) | | <.001 | 26.4 (1) | | <.001 | 102 (1) | | <.001 | 97 (1) | | <.001 |
| ANC 4+ = received four or more antenatal care visits; Coef. = Coefficient; HEW = Health extension worker; L10K = Last Ten Kilometers Project; SE = Standard error; SNNP = Southern Nations, Nationalities and Peoples; WDA = Women’s Development Army | | | | | | | | | | | | | | | | |

| Kebele-level random effects logit model coefficients predicting the outcomes of interest and goodness-of-fit statistics, continued … | | | | | | | | | | |
| --- | --- | --- | --- | --- | --- | --- | --- | --- | --- | --- |
| Independent variables |  | Immediate breastfeeding  (N=2,309) | | | Fully vaccinated  (N=4,728) | | | Dropout rate  (N=4,728) | | |
|  |  | Coef. | SE | p-value | Coef. | SE | p-value | Coef. | SE | p-value |
| Age |  | -0.023 | 0.013 | 0.064 | 0.019 | 0.008 | 0.020 | -0.019 | 0.012 | 0.098 |
| Education |  | 0.057 | 0.022 | 0.010 | 0.019 | 0.012 | 0.124 | -0.055 | 0.018 | 0.002 |
| Marital status | Other |  |  |  |  |  |  |  |  |  |
|  | In union | -0.090 | 0.352 | 0.799 | 0.317 | 0.181 | 0.080 | -0.095 | 0.272 | 0.726 |
| Number of | 1 |  |  |  |  |  |  |  |  |  |
| Children | 2 | 0.463 | 0.180 | 0.010 | 0.136 | 0.113 | 0.229 | -0.241 | 0.159 | 0.130 |
|  | 3 | 0.686 | 0.198 | 0.001 | 0.200 | 0.125 | 0.109 | -0.150 | 0.174 | 0.391 |
|  | 4+ | 0.449 | 0.210 | 0.032 | -0.056 | 0.129 | 0.667 | -0.140 | 0.180 | 0.437 |
| Religion | Orthodox |  |  |  |  |  |  |  |  |  |
|  | Protestant | -0.006 | 0.228 | 0.978 | -0.034 | 0.145 | 0.816 | 0.201 | 0.196 | 0.305 |
|  | Muslim | -0.335 | 0.238 | 0.160 | -0.043 | 0.160 | 0.788 | -0.246 | 0.224 | 0.271 |
|  | Other | -0.096 | 0.548 | 0.861 | -0.114 | 0.405 | 0.778 | -0.110 | 0.569 | 0.847 |
| Distance to any | <30 minutes |  |  |  |  |  |  |  |  |  |
| health facility | 30 min - <1 hr | -0.492 | 0.122 | <.001 | -0.063 | 0.080 | 0.430 | 0.130 | 0.112 | 0.245 |
|  | 1+ hours | -0.336 | 0.151 | 0.027 | -0.212 | 0.106 | 0.045 | 0.360 | 0.147 | 0.014 |
| Wealth quintile | Lowest |  |  |  |  |  |  |  |  |  |
|  | Second | 0.284 | 0.147 | 0.053 | -0.163 | 0.106 | 0.124 | 0.167 | 0.150 | 0.265 |
|  | Middle | 0.806 | 0.160 | <.001 | 0.085 | 0.113 | 0.451 | 0.025 | 0.157 | 0.872 |
|  | Fourth | 0.753 | 0.177 | <.001 | 0.112 | 0.120 | 0.349 | -0.103 | 0.168 | 0.541 |
|  | Highest | 0.858 | 0.203 | <.001 | 0.306 | 0.132 | 0.021 | -0.372 | 0.188 | 0.048 |
| WDA density | Lower |  |  |  |  |  |  |  |  |  |
|  | Moderate | 0.036 | 0.167 | 0.830 | 0.208 | 0.132 | 0.117 | -0.310 | 0.170 | 0.068 |
|  | Higher | 0.142 | 0.193 | 0.463 | 0.137 | 0.154 | 0.372 | -0.139 | 0.197 | 0.480 |
| HEW density | 2499 |  |  |  |  |  |  |  |  |  |
|  | 2,500 to 3,499 | -0.364 | 0.180 | 0.044 | -0.198 | 0.144 | 0.171 | 0.363 | 0.188 | 0.053 |
|  | 3,500 to 4,999 | -0.498 | 0.211 | 0.018 | -0.575 | 0.170 | 0.001 | 0.826 | 0.219 | <.001 |
|  | 5,000+ | -0.048 | 0.250 | 0.848 | -0.615 | 0.196 | 0.002 | 0.702 | 0.252 | 0.005 |
| L10K area | No |  |  |  |  |  |  |  |  |  |
|  | Yes | 0.203 | 0.190 | 0.287 | 0.285 | 0.155 | 0.065 | -0.314 | 0.199 | 0.115 |
| Region | Tigray |  |  |  |  |  |  |  |  |  |
|  | Amhara | -2.117 | 0.261 | <.001 | -0.599 | 0.176 | 0.001 | 1.569 | 0.253 | <.001 |
|  | Oromia | -1.075 | 0.349 | 0.002 | -0.446 | 0.230 | 0.052 | 1.684 | 0.326 | <.001 |
|  | SNNP | -0.992 | 0.314 | 0.002 | -0.597 | 0.211 | 0.005 | 1.550 | 0.299 | <.001 |
| Constant |  | 2.103 | 0.544 | <.001 | 0.165 | 0.341 | 0.628 | -2.653 | 0.495 | <.001 |
|  | | Statistics | | p-value | Statistics | | p-value | Statistics | | p-value |
| Global Wald’s chi-square (d.f.) | | 174 (24) | | <.001 | 78 (24) | | <.001 | 97 (24) | | <.001 |
| Random effects | | 91 (1) | | <.001 | 301 (1) | | <.001 | 184 (1) | | <.001 |
| Coef. = Coefficient; HEW = Health extension worker; L10K = Last Ten Kilometers Project; SE = Standard error; SNNP = Southern Nations, Nationalities and Peoples; WDA = Women’s Development Army | | | | | | | | | | |
